# Compliance with clinical guidelines for breast cancer management: A population-based study of quality-of-care indicators in France

**DOI:** 10.1371/journal.pone.0224275

**Published:** 2019-10-23

**Authors:** Anne Cowppli-Bony, Brigitte Trétarre, Emilie Marrer, Gautier Defossez, Laetitia Daubisse-Marliac, Gaelle Coureau, Pamela Minicozzi, Anne-Sophie Woronoff, Patricia Delafosse, Florence Molinié, for the FRANCIM network

**Affiliations:** 1 Loire-Atlantique Vendee Cancer Registry, Nantes, France; 2 SIRIC ILIAD INCa-DGOSInserm_12558, CHU Nantes, Nantes, France; 3 French Network of Cancer Registries (FRANCIM), Toulouse, France; 4 Hérault Cancer Registry, Montpellier, France; 5 Haut-Rhin Cancer Registry, Mulhouse, France; 6 Poitou-Charentes Cancer Registry, Poitiers, France; 7 Tarn Cancer Registry, Albi, France; 8 Gironde Cancer Registry, Bordeaux, France; 9 Analytical Epidemiology and Health Impact Unit, Department of Preventive and Predictive Medicine, Fondazione IRCCS Istituto Nazionale dei Tumori, Milan, Italy; 10 Doubs and Belfort Territory Cancer Registry, Besancon, France; 11 Isère Cancer Registry, Grenoble, France; Harran Universitesi, UNITED STATES

## Abstract

**Background:**

The European Society of Breast Cancer Specialists (EUSOMA), which aims to standardize the quality of patient care in Europe, has defined quality indicators (QIs) for breast cancer (BC) care to assess compliance to current care standards. These QIs are a useful tool to evaluate care organizations. Only population-based studies are able to assess health system performance in “real-life” situations. This population-based study aimed to describe compliance with several EUSOMA QIs overall and according to patient and organizational factors in France.

**Methods:**

1 560 adult women with primary invasive non-metastatic BC diagnosed in 2012 were randomly selected among all incident BC from 16 French geographical areas covered by cancer registries. Twelve EUSOMA QIs were selected regarding diagnosis, treatment and staging.

**Results:**

The minimum standard as proposed by EUSOMA was met for nine QIs related to pre-operative definitive diagnosis, multidisciplinary discussion and treatment (single surgery, breast conserving surgery (BCS) for small BC (<3cm), radiotherapy after BCS or mastectomy for regional BC (pN≥2a), hormonotherapy, adjuvant chemotherapy and trastuzumab). Low compliance was observed for sentinel lymph node biopsy (SLNB) and staging imaging. Adherence to guidelines was usually lower in older patients and in patients with comorbidities. Multidisciplinary discussion was positively related to adherence to guidelines for diagnosis, staging practices (SNLB, imaging) and systemic treatments. Compliance also varied by area of residence and by place of first treatment.

**Conclusion:**

This study provides the first current, comprehensive overview of BC quality care at a population level in France. The guidelines were correctly applied in percentage satisfying the EUSOMA standards for the diagnosis and treatment of BC, although staging practices (SLNB, imaging) can be improved. These results highlight the need for continuous measurement of adherence to guidelines to improve BC care.

## Introduction

Breast cancer (BC) is the most commonly diagnosed cancer and leading cause of cancer death in women worldwide [1]. In France, 52621 women were diagnosed with BC in 2012, resulting in 11 780 deaths according to national estimates from cancer registries [2].

To ensure optimal BC care for all patients, clinical practice guidelines have been developed by health organizations and oncology societies [3]. The European Society of Breast Cancer Specialists (EUSOMA), which aims to standardize the quality of patient care in Europe, has defined 33 quality indicators (QIs) to assess compliance to current care standards [4]. Similar QIs have been defined in the US [5]. These indicators may be useful for identifying gaps and areas for quality improvement at local and national levels.

In France, cancer care is delivered by multiple providers, including private fee-for-service physicians, public hospitals and private (non-profit-making and profit-making) hospitals. Each patient is free to choose his physician and his health care facility for care and benefits from a full medical coverage for cancer guaranteeing free access to cancer care.

Population-based cancer registries provide non-biased information on cancer management and are potentially able to assess health system performance, especially regarding the application of guidelines. To our knowledge, no population-based study on QIs for BC management has been conducted in France.

The present population-based study aimed to assess the quality of non-metastatic BC management in France by evaluating compliance with several EUSOMA QIs overall and according to patient and organizational factors. We also described reasons for non-compliant practices.

## Materials and methods

### Population

Data were provided by all French population-based cancer registries of the FRANCIM network, covering 22% of the French population: Bas-Rhin, Calvados, Côte d’Or, Doubs, Haut-Rhin, Haute-Vienne, Hérault, Gironde, Isère, Lille area, Loire-Atlantique, Manche, Poitou-Charentes, Somme, Tarn and Vendée. FRANCIM cancer registries record all new cases of cancer from residents in a geographic area (departement, region or city). The quality and completeness of registry data are certified every five years by the national Evaluation Committee of Registries.

Approximately 100 adult women (≥18 years) with primary BC diagnosed in 2012 were randomly sampled from each registry to compile a large representative sample (*n* = 1 855). Regarding the sampling procedure, each registry first selected days and months of birth and then patients born those days were systematically included in the study. Sampled patients represented 13% of all the BC women diagnosed in 2012 in FRANCIM registries. Each cancer registry contributed equally (i.e. regardless of the size of the geographical area covered) to the study sample.

Only women with carcinomas were included. Patients who presented prior in situ or invasive breast carcinoma were also excluded. This study considered 1 560 women with non-metastatic invasive BC after excluding in situ and metastatic cancers.

### Data collection

In addition to data routinely collected by registries, extensive information was collected from medical records: mode of detection, comorbidities, tumor characteristics at diagnosis (clinical and pathological TNM stages, Scarff-Bloom-Richardson (SBR) grade, estrogen (ER) and progesterone (PR) receptor status and human epidermal growth factor receptor-2 (HER2) status), staging imaging and therapeutic management.

According to TNM Classification of Malignant Tumours (7th edition), stage was defined from pathological stage or from clinical stage in case of neoadjuvant or non-surgical treatment. A phenotypic subtype was defined with hormonal and HER2 status according to the international classification on molecular subtypes of BC [6].

### Outcome measures

Twelve QIs were selected considering various aspects of care: diagnosis (pre-operative diagnosis (QI_3b)), treatment (multidisciplinary discussion (QI_8), appropriate surgical approach (QI_9a), post-operative radiotherapy (QIs_10a/b), avoidance of overtreatment (QIs_11a/c), and appropriate systemic treatment (QIs_12_13a/b)) and staging procedures (QIs_14a/b) [4]. The most relevant QIs were selected based on the purpose of the study and available data. The last updated (2017) EUSOMA QIs [4] were used instead of QIs from 2010 [7]. The QIs selected have similar definitions between 2010 and 2017 and reflect the French 2012 guidelines.

### Statistical analysis

The outcome measure was the proportion of patients treated in accordance with guidelines for each indicator with its 95% confidence interval (CI). Appropriate patient selection was used for each QI. Patients with missing data on the variables of interest for a specific indicator were excluded from analyses. Considering the complex management of elderly patients, EUSOMA recently proposed the exclusion of older patients (≥70) from analyses in case of low adherence to QIs_10_12_13 [4]. Only QI_13a required application of this recommendation in this study.

Compliance with each QI was compared between patient groups stratified by age (<50, 50–74, and ≥75 years), Charlson comorbidity index [8] (characterized as 0, 1 or ≥2), place of first treatment delivery (grouped into four categories: comprehensive cancer centers (CCC), teaching hospitals (TH), public and private hospitals), multidisciplinary discussion and geographical area of residence covered by the registries. Comparisons of compliance between groups were made using two-sided Chi-square or Fisher’s exact tests (in case of low numbers). Analyses were performed using STATA/IC 14.

### Ethics statement

All FRANCIM cancer registries are approved by the French National Commission on Information Technologies and Liberties (CNIL) to collect nominative data on cancer patients without informed consent, for research purposes and in the strictest confidentiality. However, each cancer patient living in a geographic area covered by a registry is informed that his data can be recorded in the registry database and that he can oppose this registration. Only fully anonymized data are published.

## Results

The characteristics of 1 560 women are presented in Table 1 (mean age = 61.2 years; range = 22–95). Women were often diagnosed at stage I and underwent first-line surgery (86.3%), mainly breast conserving surgery (BCS) (74.0%).

**Table 1:** Characteristics of patients diagnosed with non-metastatic invasive breast cancers in 2012 in France (N = 1 560).

Characteristics	*N*	*%*
**Age at diagnosis (years)**		
< 50	340	21.8
50–74	933	59.8
≥ 75	287	18.4
**Charlson comorbidity index**		
0	1 128	72.3
1	232	14.9
≥2	186	11.9
Unknown	14	0.9
**Mode of detection**		
Organized/opportunistic screening	793	50.8
Clinical diagnosis	730	46.8
Other	22	1.4
Unknown	15	1.0
**SBR grade**		
1	384	24.6
2	781	50.1
3	366	23.5
Unknown	29	1.9
**Phenotypic subtype**		
Luminal A/B (ER+ and/or PR+, HER2 -)	1 198	76.8
Luminal A/B-HER2 (ER+ and/or PR+, HER2 +)	120	7.7
HER2 (ER-, PR-, HER2+)	78	5.0
Triple negative (ER-, PR-, HER2-)	148	9.5
Undetermined	16	1.0
**Stage at diagnosis**		
IA (T1-N0-M0)	755	48.4
IB (T0/1-N1mi-M0)	42	2.7
IIA (T0/1-N1-M0 or T2-N0-M0)	366	23.5
IIB (T2-N1-M0 or T3-N0-M0)	196	12.6
IIIA (T0/1/2-N2-M0 or T3-N1/2-M0)	95	6.1
IIIB (T4-N0/1/2-M0)	57	3.6
IIIC (anyT-N3-M0)	32	2.1
Unknown (T or N missing, M0)	17	1.1
**Treatment**		
Surgery (+/- HT)	111	7.1
Surgery with adjuvant RT (+/- HT)	695	44.6
Surgery with adjuvant CT + RT (+/- HT)	496	31.8
Surgery with adjuvant CT (+/- HT)	43	2.8
Neoadjuvant therapy (CT or HT) and surgery	140	9.0
Other treatments (CT, RT, HT) without surgery	58	3.7
None	14	0.9
Unknown	3	0.2
**Place of first treatment delivery**		
Public hospital	222	14.2
Private hospital	708	45.4
Teaching hospital	161	10.3
Comprehensive cancer center	399	25.6
Unknown	70	4.5

SBR, Scarff-Bloom-Richardson (SBR); ER, estrogen receptor; PR, progesterone receptor; HER2, human epidermal growth factor receptor-2; CT, chemotherapy; RT, radiotherapy; HT, hormonotherapy.

As shown in Table 2, nine QI related to diagnosis and treatment achieved the minimum EUSOMA standards, whereas three QI for compliance were lower than the minimum standards.

**Table 2:** Definition of EUSOMA quality indicators and compliance in 1 560 patients diagnosed with non-metastatic invasive breast cancers in 2012 (France).

Definition of EUSOMA QIs		Minimum / target standards (%)	Number of eligible cases	Compliance			Missing	
				*N*	*%*	*(95% CI)*	*N*	%
***Diagnosis***								
Pre-operative diagnosis	3b. Proportion of women who had a pre-operative histologically or cytologically definitive diagnosis	85 / 90	1 560	1 522	97.6	(96.7–98.3)	0	
***Surgery and locoregional treatment***								
Multidisciplinary discussion	8. Proportion of patients to be discussed by a multidisciplinary team	90 / 99	1 560	1 491	97.6	(96.7–98.3)	32	2.1
Appropriate surgical approach	9a. Proportion of patients with surgical treatment who received a single (breast) operation for the primary tumor (excluding reconstruction)	80 / 90	1 485	1 300	87.5	(85.8–89.2)	0	
Post-operative radiotherapy	10a. Proportion of patients who received post-operative radiotherapy after surgical resection of the primary tumor and appropriate axillary staging/surgery in the framework of BCS	90 / 95	969	945	97.8	(96.7–98.6)	3	0.3
	10b. Proportion of patients with involvement of axillary lymph nodes (≥pN2a) who received postmastectomy radiotherapy	90 / 95	59	55	93.2	(83.5–98.1)	0	
Avoidance of overtreatment	11a. Proportion of patients with a clinically negative axilla (cN0) who had sentinel lymph node biopsy only	90 / 95	1 175	895	76.2	(73.7–78.6)	1	0.1
	11c. Proportion of patients with BC not greater than 3 cm who underwent BCS as primary treatment	70 / 85	1 172	947	80.8	(78.5–83.1)	0	
***Systemic treatment***								
Appropriate endocrine therapy	12. Proportion of patients with endocrine-sensitive BC who received endocrine therapy	85 / 90	1 318	1 215	93.0	(91.5–94.4)	12	0.9
Appropriate chemotherapy and HER2-targeted therapy	13a. Proportion of patients with ER–(T > 1 cm or N+) BC who received adjuvant chemotherapy	85 / 95	139	114	82.6	(75.2–88.5)	1	0.7
		*(<70 years)*	*96*	*92*	*95*.*8*	*(89*.*7–98*.*9)*	*0*	
	13b. Proportion of patients with HER2+ (T > 1 cm or N+) BC treated with chemotherapy who received adjuvant trastuzumab	85 / 95	104	101	98.1	(93.2–99.8)	1	1.0
***Staging*, *counseling*, *follow-up and rehabilitation***								
Appropriate staging procedure	14a. Proportion of women with stage I or primary operable stage II BC who do not undergo baseline-staging tests	95 / 99	1 359	325	24.7	(22.4–27.1)	44	3.2
	14b. Proportion of women with stage III BC who undergo baseline-staging tests	95 / 99	184	144	79.1	(72.5–84.8)	2	1.1

Patients with missing values were excluded to calculate compliance per QI. The proportion of missing values indicates the missing values of the variables of interest in the selection that was made for the specific QI.

BC, breast cancer; BCS, breast conserving surgery; ER, estrogen receptor; T, tumor size; N, node involvement.

Very high adherence to recommendations (above target standards) was found for five QIs (QIs_3b_10a_12_13a/b). The reasons for non-compliance are described below.

- *QI_3b (pre-operative histological/cytological diagnosis)*: Of 38 women without pre-operative diagnosis (non-compliance rate of 2.4%), 34 women underwent surgery as first-line treatment. Four women had no histological diagnosis or biopsy after first hormonal treatment.
- *QI_10a (radiotherapy after BCS) and* QI_12 (endocrine therapy): Non-compliance (2.2% and 7.0% respectively) was mostly due to physician or patient choice (Table 3).
- *QI_13a (adjuvant chemotherapy)*: Despite general low adherence (82.6%), adherence became very high (95.8%) when older patients (≥70 years) were excluded. Chemotherapy was often not performed because of medical choice or contraindication (Table 3).
- *QI_13b (adjuvant trastuzumab)*: Only two women (ages 45 and 68 years old) did not receive adjuvant trastuzumab among 104 eligible cases (non-compliance rate of 1.9%).

**Table 3:** Reasons for non-compliance with treatment QIs in patients diagnosed with non-metastatic invasive breast cancers in 2012 (France).

Reasons for non-compliance	QI_10a		QI_10b		QI_12		QI_13a	
	*N*	*%*	*N*	*%*	*N*	*%*	*N*	*%*
Patient refusal	8	36.4	1	25.0	28	30.8	2	8.3
Medical choice	7	31.8	0	0.0	44	48.4	13	54.2
Contraindication	4	18.2	1	25.0	8	8.8	7	29.2
Death	1	4.5	2	50.0	4	4.4	1	4.2
Other (another synchronous cancer)	2	9.1	0	0.0	3	3.3	0	0.0
Unknown	0	0.0	0	0.0	4	4.4	1	4.2
Total of non-compliant cases	22	100.0	4	100.0	91	100.0	24	100.0

High adherence to recommendations (between minimum and target standards) was observed for four QIs:

- *QI_8 (multidisciplinary discussion)*: Medical files of 37 women were not examined within a multidisciplinary team meeting (MTM) (non-compliance rate of 2.4%). These women received mainly hormonotherapy alone (48.6%), whereas the others had surgery (alone (8.1%) or with adjuvant treatments (24.3%)), neoadjuvant therapy (2.7%) or no treatment (16.2%).
- *QI_9a (single surgery)*: A total of 185 women underwent several breast operations (non-compliance rate of 12.5%): 164 had a second operation (half were BCS), whereas 21 had three surgeries.
- *QI_10b (postmastectomy radiotherapy for BC with pN≥2a)*: Four women (non-compliance rate of 6.8%) did not have radiotherapy because of patient factors (Table 3).
- *QI_11c (BCS for small BC*, *defined as histological tumor size <30mm)*: Among 225 women with small BC who underwent mastectomy (non-compliance rate of 19.2%), 28.0% had a first BCS with positive margins and 58.2% had a multicentric or overlapping tumor, which is an indication to perform mastectomy.

Low adherence to recommendations was observed for three QIs:

- *QI_11a (sentinel lymph node biopsy (SLNB))*: Among 279 patients not receiving SNLB (non-compliance rate of 23.8%), 208 had an axillary lymph node dissection (ALND). Other patients received surgery without axillary procedure (*n* = 21), hormonotherapy alone (*n* = 41) or no treatment (*n* = 9). Compared to compliant cases, non-compliant BCs with ALND had higher T-stage (44.7/38.5% T1/T2 vs 82.9/14.3%), grade (32.2% grade 3 vs 18.0%) and more aggressive phenotypes (5.0/10.6% HER2/triple negative vs 3.0/7.8%).
- *QI_14a (staging procedures for stages I-II)*: Contrary to guidelines, 75.2% of patients at stage I-II underwent at least one test (stage I: 65.8% and stage II: 88.7%). Chest, abdomen and bone were all explored for 72.1% of patients.
- *QI_14b (staging procedures for stage III)*: Only 79.1% of patients at stage III underwent complete work-up. The 38 non-compliant cases had no (7.9%) or incomplete work-up (15.8% and 76.3% with one and two tests, respectively). Most patients (*n* = 35) received treatments (surgery, hormonotherapy alone or neoadjuvant therapy).

**Fig 1:**
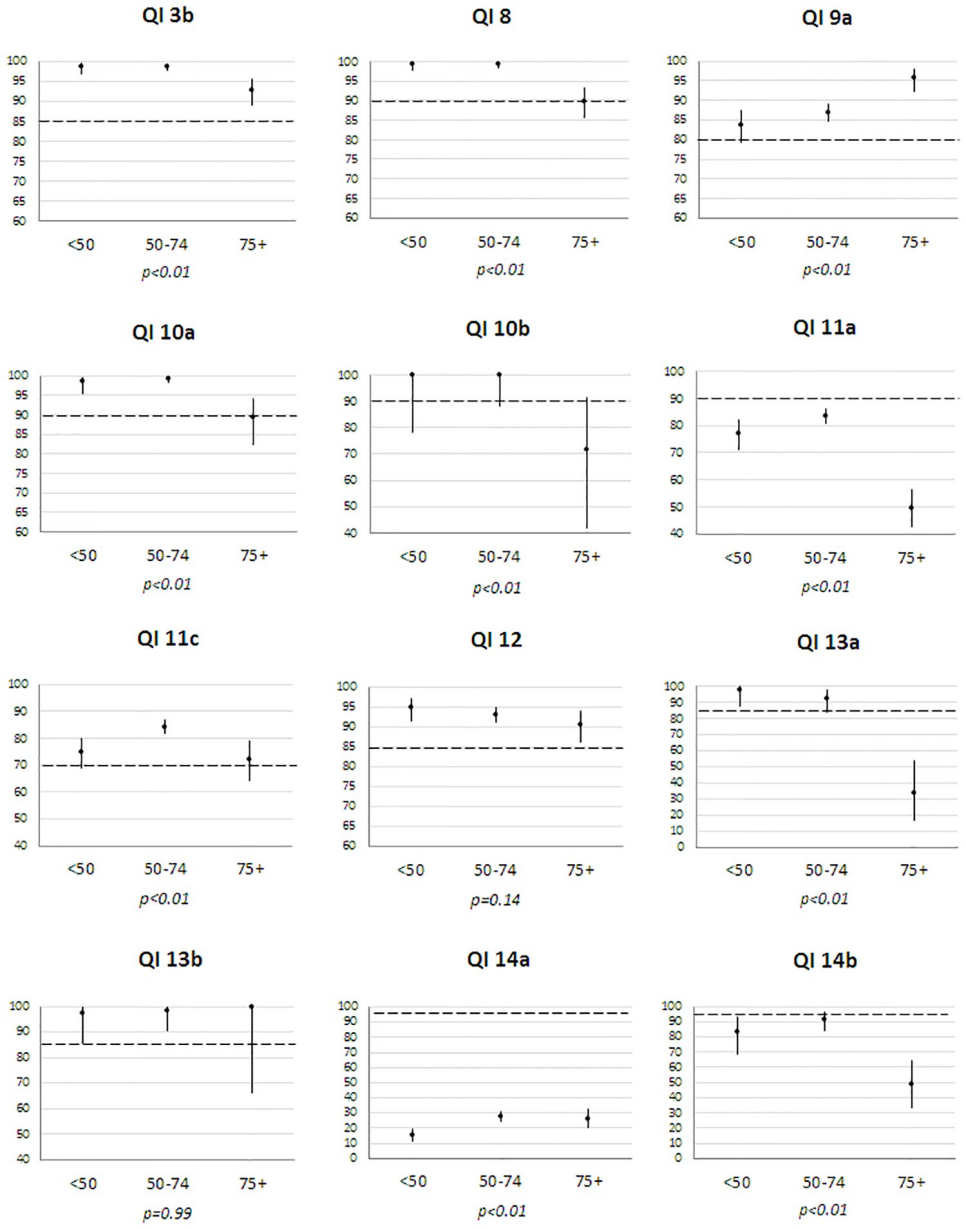
Compliance (%) of each QI by age group (<50, 50–74, ≥75 years) in 1 560 patients diagnosed with non-metastatic invasive breast cancers in 2012 (France). The extent of the Y scale for compliance (%) is different across QIs. The dotted line represents the minimum standard for each QI. *p*: Fisher tests were used for QIs 10a/b and 13a/b.

**Fig 2:**
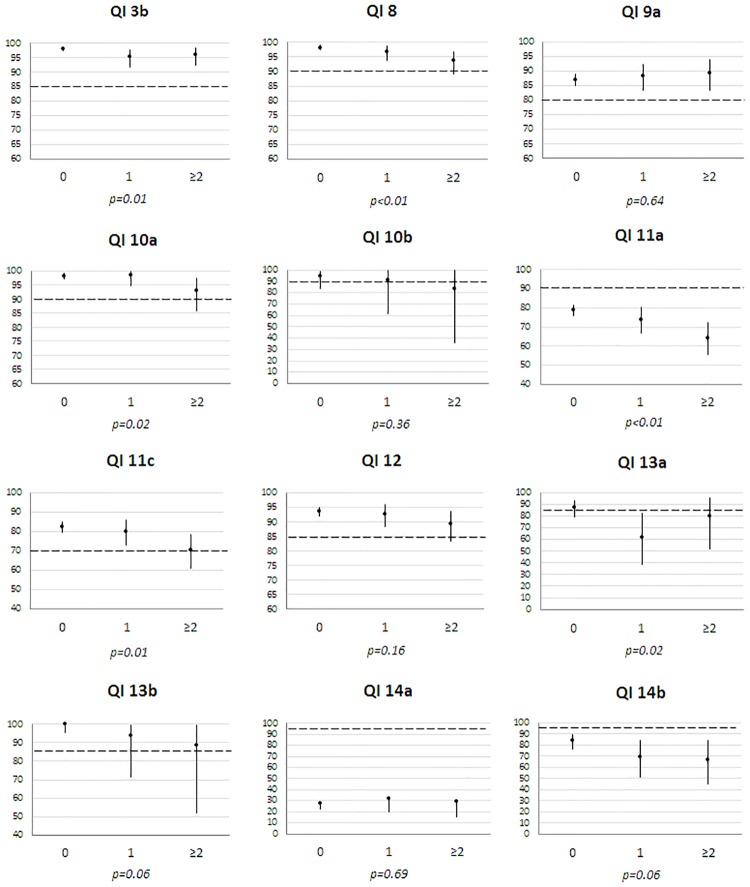
Compliance (%) of each QI by Charlson comorbidity index in 1 560 patients diagnosed with non-metastatic invasive breast cancers in 2012 (France). The extent of the Y scale for compliance (%) is different across QIs. The dotted line represents the minimum standard for each QI. *p*: Fisher tests were used for QIs 10a/b and 13a/b.

**Fig 3:**
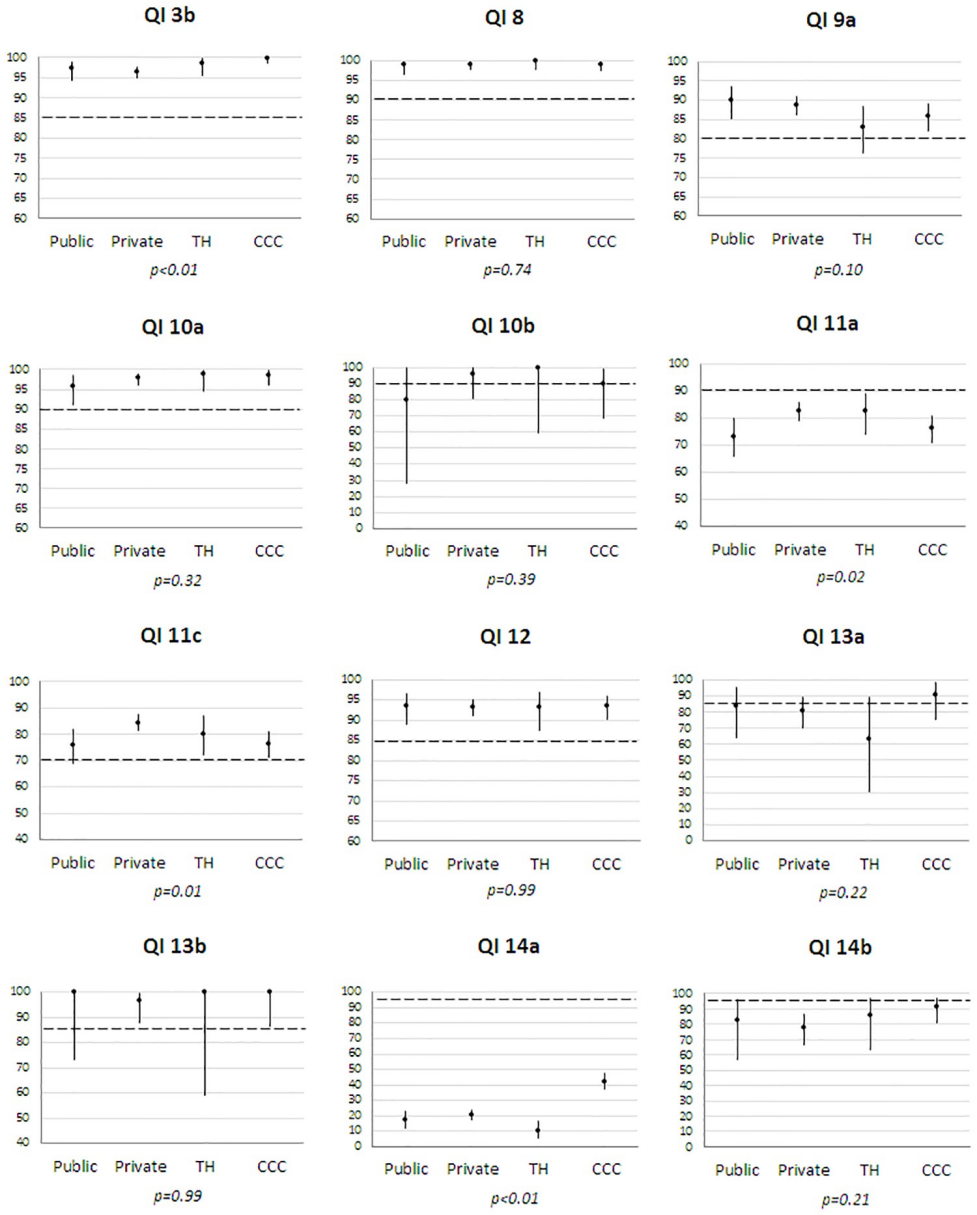
Compliance (%) of each QI by place of first treatment delivery (public and private hospitals, teaching hospital (TH) and comprehensive cancer center (CCC)) in 1 560 patients diagnosed with non-metastatic invasive breast cancers in 2012 (France). The extent of the Y scale for compliance (%) is different across QIs. The dotted line represents the minimum standard for each QI.*p*: Fisher tests were used for QIs 3b, 8, 10a/b, 11a, 12, 13a/b and 14b.

Regarding compliance by age (Fig 1), older patients (≥75) often had significantly lower adherences, except for QIs_12_13b_14a. Higher adherence was observed among older patients for single breast operation (QI_9a), whereas BCS for small BC (QI_11a) was more frequent in the 50–74 age group. Adherence was often higher in patients without comorbidity except for QIs_9a_14a (Fig 2). When medical files were examined within a MTM, compliance was better for preoperative diagnosis (QI_3b: 97.8% vs 89.2% p-Fisher = 0.01), SLNB (QI_11a: 78.0% vs 25.8% p<0.01), hormonotherapy (QI_12: 93.4% vs 83.3% p-Fisher = 0.049) and staging tests for stage III (QI_14b: 81% vs 0%, p-Fisher<0.01). All patients receiving chemotherapy (QI_13a/b) were discussed at MTM. No compliance difference was found for surgery (QI_9a_11c), radiotherapy (QI_10a/b) and staging tests for stage I-II (QI_14a). Compliance differed by place of first treatment for four QIs (Fig 3). It was higher in CCC for pre-operative diagnosis (QI_3b) and staging tests for stage I-II (QI_14a), lower for SLNB in public hospitals (QI_11a) and higher for BCS for small BC in private hospitals (QI_11c). Compliance also varied by geographical area of residence for single surgery, SLNB, BCS, hormonotherapy and staging tests for stage I-II (QIs_9a_11a/c_12_14a) (S1 Fig).

## Discussion

The guidelines were correctly applied in percentage satisfying the EUSOMA standards in France for QIs related to diagnosis and treatment of invasive BC. Low adherence to recommendations was observed for SLNB and staging imaging. Adherence to guidelines was usually lower in older patients and patients with comorbidities. Compliance also varied by area of residence, place of first treatment and MTM.

Numerous studies have examined BC care quality. Several studies were conducted before 2003, when guidelines and treatments differed than those currently in use. Out of all recent studies [9–29], the only few conducted at the population level [9–12,16,17,28,29] used recent data (after 2010) [28,29]. Five studies using EUSOMA QIs [22,24–27] were hospital-based and included patients from voluntary centers (often EUSOMA certified centers [22,24–26]), which may introduce selection biases and overestimate compliance. The assessment of health system performance in BC care requires measuring of compliance in “real-life” situations. In 2019, two population-based studies on EUSOMA QIs for the management of BC diagnosed in 2013 and 2016 were conducted in Slovenia and Norway [28,29]. The results of these studies are difficult to generalize to French patients because of differences between health systems. The French system presents some distinguishing features: the importance of private sector which is accessible to all insured patients, complete freedom of provider choice and no limitation of utilization of services.

Regarding pre-operative histological/cytological diagnosis, similar high compliance rates (93–98%) were observed in four recent European studies [20,21,23,29], while two studies that included in situ cancers found lower rates (86–88%) [22,26]. Another French study found perfect compliance, probably due to patient selection (i.e., operable early-stage BC patients managed in CCC, TH and general hospitals) [27]. Older studies found lower compliance rates (60–70%) [10,11,17], except for one study that excluded elderly patients [12].

The guidelines were correctly applied in percentage satisfying the EUSOMA standards for surgical procedures (single operation and BCS for small BC). In most European studies, reexcision rates were similar to ours [10,11,19,25,26], while American studies reported higher rates (25–40%) [30]. The Norwegian study reported lower reexcision rate (6%) in 2016 [29], in line with the rate decrease observed in another study between 2012 (14.6%) and 2015 (8.8%) [26]. For BCS, variation in compliance was found between studies and may be explained by stage and age differences in the analyzed populations. Compliance rates were similar (81–86%) in European studies that have the same design as ours [22,25,26,29], except for the Slovenian study which reported a low rate (67.5%) [28]. Several American studies reported also low rates (60–70%) in early-stage BCs [31–35]. In our study, 20% of women with small BC underwent mastectomy. In most of them, mastectomy was used to achieve free margins after BCS or was justified by tumor characteristics. Another primary reason for choosing BCS or mastectomy is patient preference. Radiotherapy was examined after BCS (QI_10a) and after mastectomy for pN≥2a (QI_10b). We reported high compliance (97.8%) for QI_10a. The recent hospital-based European studies found similar rates (94–98%) [22,23,25,26] while lower rates (92–93%) were observed in the Norwegian and Slovenian population-based [28,29]. Older studies found also lower rates [11–13,15,17,20]. In the US, radiotherapy after BCS was less frequent (80%) with geographic disparities [16,34,35]. For QI_10b, the minimum standard (90%) was reached in our study contrary to the two studies reporting low compliance (85.2% in 2008–2012 [25] and 89.9% in 2013 [28]). For these two QIs, non-compliance was mainly due to patient factors in our study.

Regarding hormonotherapy (QI_12a), our results were concordant with previous studies [10,13–15,20,22,23,25,26,28]. Non-compliance was related to patient factors or physician decision based on tumor characteristics (very early-stage, weak hormone receptor-positivity) and the harm/benefit ratio. This is in contradiction with guidelines which recommend hormonotherapy for all endocrine-positive BC, except for small BC (≤T1aN0) [3,36]. Adherence to recommendations for adjuvant chemotherapy (QI_13a) was generally low (83%) but became very high after elderly patients were excluded (96%). This low compliance may also be explained by the preponderance of BC with aggressive phenotypes (triple negative or HER2+) in the eligible population. Neoadjuvant chemotherapy is recommended for these cancers. Two studies reported similar compliances (83–85%) [26,28], while higher rates (90–96%) were seen in the other studies, probably due to exclusion of elderly populations and study design [14,22,24,25]. Compliance with trastuzumab use (QI_13b) was also high in our study. Only the Slovenian study examined this QI and reported a lower rate (87.7%) [28].

The minimum EUSOMA standard was not met for staging practices. For SLNB, low adherence was seen in our study (76%), which is in line with recent European hospital-based studies with similar methods (81–82%) [24,25]. Like an American hospital-based study finding a higher compliance rate (87%) in 2009 [18], a higher compliance rate (89.5%) was observed in Slovenia in 2013 [28], probably due to the fact that BC management is centralized. ALND was chosen in place of SLNB in 17.5% of eligible women, whereas the others did not have surgery. SLNB is indicated for staging patients with early BC (T1-T2N0) [3,37]. In France, this recommendation is restricted to T1-T2 (≤30mm) BC [36]. When tumor size (≤30mm) was considered, compliance remained low at 81.0%. Thus, this low adherence mainly indicates a possible aggressive treatment approach, even if some prognostic factors (such as multifocality/multicentricity, high grade, and triple negativity) may have influenced the choice of this approach. Besides SLNB helps to choose the best treatment for each patient, it reduces the chance of related arm morbidity, common after ALND. Moreover, guidelines were not followed for metastatic work-up: imaging was performed too often in early BC (stages I-II) and not enough often in stage III BC. These observations have already been reported in several studies [17,38,39]. Guidelines recommend imaging only for patients with symptomatic early BC and stage III BC because the reported probability of occult distant metastasis in stage I-II BC is exceedingly low (0.3–1.2%) [3,4,38]. In our study, we could not distinguish between asymptomatic and symptomatic BC, which might limit our interpretations. However, non-compliance was very high and could not entirely be explained by symptomatic women. Non-compliance could be explained by patient- and physician-related factors [40,41]. Physician behavior in ordering unnecessary tests might be partly driven by patient demand [40]. Fear of malpractice litigation may be another explanation. Given the cost and morbidity associated with unnecessary tests, patient and physician education regarding performing appropriate tests is required.

Finally, we found high adherence for MTM. The other French study also observed high compliance (94–95%), except for TH (32%) [27]. Although MTM has become standard in BC management in many countries worldwide, few studies have examined how many patients benefit from it. Stordeur et al. found an 80.3% compliance rate in 2006 [11]. Kowalsky et al. found rates from 58.3% (pre-treatment MTM) to 100% (post-operative MTM) in 2012 [23]. In our study, MTM was positively related to high-quality care in line with guidelines, especially the confirmation of the malignant diagnosis—which is required for ascertaining the optimal treatment -, the choice of the appropriate systemic treatments and SNLB use, a more recent staging practice.

Elderly patients were less likely to receive care that conformed to guidelines, which was consistent with previous studies [16,17,25,42]. These results may reflect undertreatment but can be related to patient preferences [43]. However, strong evidence exists that more conservative approaches to surgery and post-operative radiotherapy may be adopted in older patients without affecting survival [44–46]. Similarly, systemic therapies should be adapted to health status and harm/benefit ratio. This result may partly explain why EUSOMA has recently proposed the exclusion of older patients when adherence fails to meet the minimum standard for some QIs [4]. The same conclusions can be drawn regarding lower compliance in patients with comorbidities, who are often elderly [17,42,47]. Variation in treatment compliance between type of heath care facilities has already been reported [17,27,48]. It may be related not only to patient and tumor characteristics (age, stage) but also to hospital and physician characteristics. Indeed, surgical procedures can vary with the medical practices of each physician, team habits or organizational factors. Territorial differences partly reflect differences in health care provisions between departments (i.e., variation in screening, distribution of hospital categories, access to some treatments such radiotherapy, and coordination care). They may also be explained by patient characteristics and physician preferences. However, minimum adherence levels should be met regardless of patient recruitment or the geographical location of health care organizations.

The main strengths of this study include its population-based design, which allowed assessment of quality care without selection bias, and data quality (few missing data). The sample procedure ensures that our sample is representative of a large population of BC patients and allows to describe the heterogeneity of medical practices in France.

However, the small number of cases for certain QIs may limit interpretations. Quality care analysis requires assessments of reasons for non-compliance because guideline may not be applied for every patient. We evaluated these reasons for most QI, except for BCS and trastuzumab. Following EUSOMA [4], our study analyzed non-metastatic BC. Complementary analyses including all invasive BC showed similar results.

## Conclusions

Our study provides the first current, comprehensive overview of BC quality care in France at a population-level. The guidelines were correctly applied in percentage satisfying the EUSOMA standards for the diagnosis and treatment of non-metastatic BC, although staging practices (SLNB, imaging) can be improved. Measurement of indicators is the starting point for understanding how to improve practices. This study may contribute to updating guidelines and can be used as baseline information prior to assessing the current national Cancer Plan.

## Supporting information

S1 Fig Compliance (%) of each QI by geographical area of residence covered by registries in 1 560 non-metastatic invasive breast cancers diagnosed in 2012 (France). The extent of the Y scale for compliance (%) is different across QIs. The dotted line represents the minimum standard for each QI. The geographical areas are represented by official area codes. Compliance range is defined as the difference between the maximum and minimum compliances for each QI. *p*: Fisher tests were used for all QIs except QI_11c.
(TIF)
