# Supplementary figures and images for: Compliance with clinical guidelines for breast cancer management: A population-based study of quality-of-care indicators in France
Source: PLoS One. 2019 Oct 23;14(10):e0224275. doi: 10.1371/journal.pone.0224275 (PMC6808419; doi:10.1371/journal.pone.0224275)

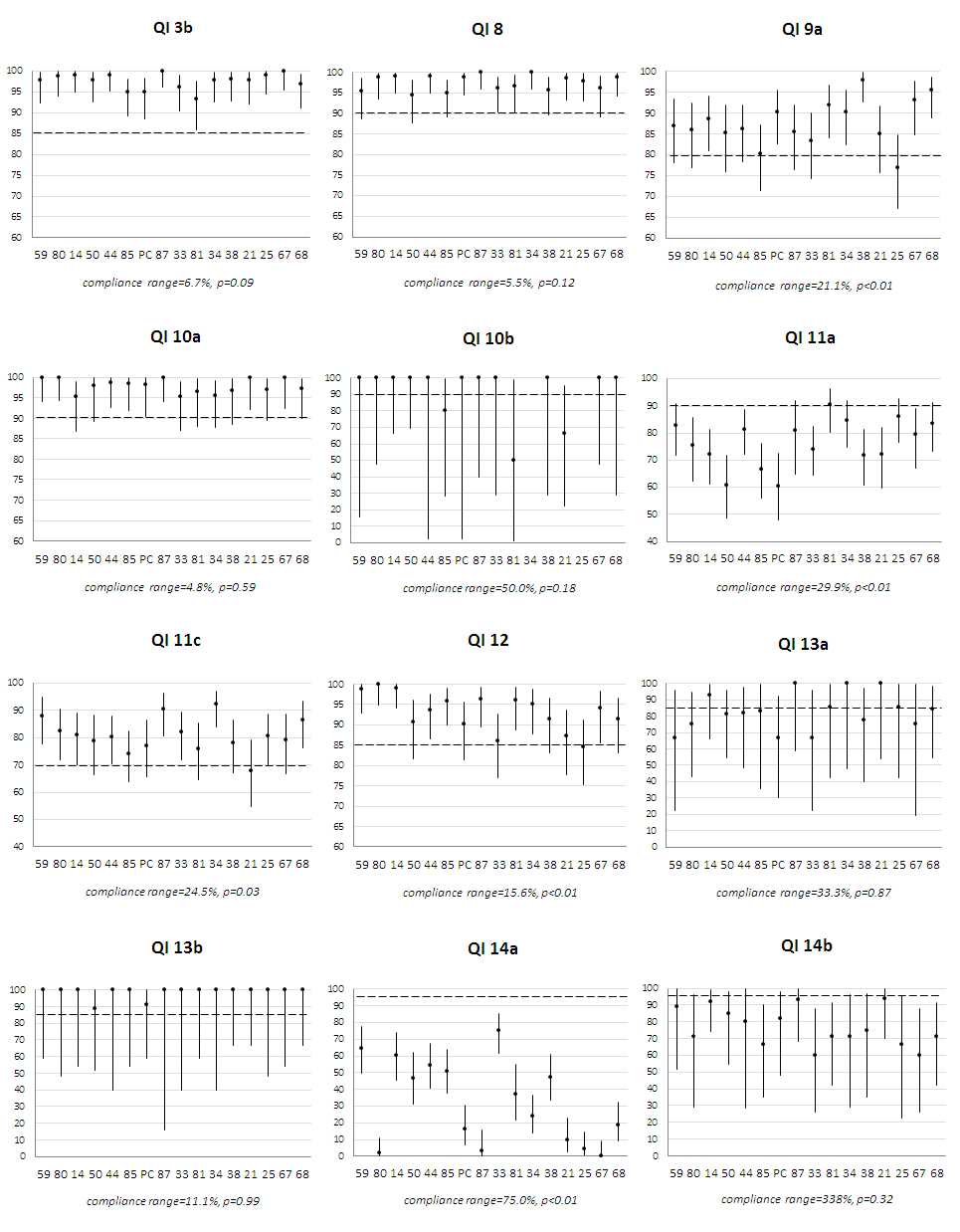

Supplement: S1 Fig — The extent of the Y scale for compliance (%) is different across QIs. The dotted line represents the minimum standard for each QI. The geographical areas are represented by official area codes. Compliance range is defined as the difference between the maximum and minimum compliances for each QI. p: Fisher tests were used for all QIs except QI_11c. (TIF) [file pone.0224275.s001.tif]
